# Promoter Characterization and Role of cAMP/PKA/CREB in the Basal Transcription of the Mouse ORMDL3 Gene

**DOI:** 10.1371/journal.pone.0060630

**Published:** 2013-04-05

**Authors:** Li-Li Zhuang, Rui Jin, Liang-Hua Zhu, Hua-Guo Xu, Yue Li, Shan Gao, Jia-Yin Liu, Guo-Ping Zhou

**Affiliations:** 1 Department of Pediatrics, The First Affiliated Hospital, Nanjing Medical University, Nanjing, Jiangsu Province, China; 2 Department of Laboratory Medicine, The First Affiliated Hospital, Nanjing Medical University, Nanjing, Jiangsu Province, China; 3 State Key Laboratory of Reproductive Medicine, Clinical Center of Reproductive Medicine, The First Affiliated Hospital, Nanjing Medical University, Nanjing, Jiangsu Province, China; Kyushu Institute of Technology, Japan

## Abstract

Orosomucoid 1-like 3 (ORMDL3) gene was strongly linked with the development of asthma in genetic association studies, and its expression could be significantly induced by allergen in airway epithelial cells of mice. However, the expression mechanism of ORMDL3 was still unclear. Here we have identified and characterized the mouse ORMDL3 gene promoter. Deletion constructs of the 5′ ﬂanking region were fused to a luciferase reporter gene. After transient transfection in mouse fibroblast cell line NIH3T3, a CRE (−27/−20) binding CREB was identified in the core promoter region. Deletion or mutation of the CRE consensus sequence resulted in a significant loss of the promoter activity. EMSA and ChIP assays demonstrated the binding of CREB to the core promoter. Knocking down endogenous CREB led to a reduction in ORMDL3 expression. Conversely, overexpression of CREB up-regulated ORMDL3 expression. Moreover, forskolin, a PKA activator, could facilitate the phosphorylation of CREB, which in turn heightens ORMDL3 expression. H-89, a PKA-specific inhibitor, could significantly inhibit ORMDL3 expression. This study delineates the characterization of mouse ORMDL3 gene promoter and shows signaling pathway cAMP/PKA/CREB plays an important role in regulating ORMDL3 expression, which will be helpful for future animal model studies regarding the regulation or function of ORMDL3 gene.

## Introduction

The orosomucoid 1-like 3 (ORMDL3) gene, which belongs to a novel evolutionarily conserved gene family (ORMDL1-3), encodes a protein locate in the endoplasmic reticulum (ER) membrane [1]. It has been well established by a series of independent genome-wide association studies (GWAS) that ORMDL3 is a risk factor for the development of many immune-related diseases, e.g. asthma, recurrent wheeze, ulcerative colitis, ankylosing spondylitis, type 1 diabetes and rheumatoid arthritis [2], [3], [4], [5], [6], [7]. Among these diseases, the relationship between ORMDL3 and asthma is best established: 1) the relationship between asthma risk alleles on 17q21 locus and ORMDL3 mRNA level is strong, and already exist in cord blood [8]; 2) changed transcript level of ORMDL3 is found in Epstein-Barr virus transformed lymphoblastoid cell lines from children with asthma [2]; 3) ORMDL3 expression can be significantly induced by allergen in airway epithelial cells of mice [9]; 4) the induction of ORMDL3 high expression in normal human lung fibroblasts by polyinosine-polycytidylic acid indicates the role ORMDL3 may play in viral respiratory infection [10], [11], a pathological state believed to induce and exacerbate asthma [12]; 5) transcript level of ORMDL3 in children with recurrent wheeze is higher than in normal children [13].

As orthologues of the yeast Orm proteins (Orm1/2), which are regulators of sphingolipid biosynthesis, the mammalian ORMDL proteins (ORMDL1/2/3) play a relevant role in maintaining the sphingolipid homeostasis. It has been reported that all the three isoforms of ORMDL proteins take part in the modulation of ceramide synthesis by directly and negatively regulating serine palmitoyltransferase (SPT) activity, the first and rate-limiting enzyme in sphingolipid production [14]. Several studies support a role for sphingolipids such as ceramide in asthma-associated inflammatory processes, including mast cell degranulation, airway hyper-responsiveness and immune-cell trafficking [15]. Besides, as an ER-resident transmembrane protein, ORMDL3 alters ER-mediated Ca^+^ homeostasis and facilitates the unfolded-protein response (UPR), a process considered as an endogenous inducer of inﬂammation, by interacting with the sarco-endoplasmic reticulum Ca^+^ pump (SERCA) [16]. The above two biological function of ORMDL3 raise the testable hypothesis that misregulation of ORMDL3 may play a causative role in the development of asthma and other ORMDL3 associated diseases partly through effecting the sphingolipid homeostasis and ER homeostasis. Elucidating the molecular mechanism that control transcription of ORMDL3 gene may yield insights into the mechanism how misregulation of ORMDL3 leads to the pathology of asthma.

Prior studies revealed that both human and mouse express the same three ORMDL family members with ORMDL3 exhibiting 96% identity between man and mouse [1]. Nevertheless, the transcriptional regulation mechanisms of different species of the same gene may not be alike. Our group has recently reported that transcriptional activity of human ORMDL3 gene was cooperatively regulated by transcription factors Ets-1, p300 and CREB through binding to the promoter of ORMDL3 gene [13]. Here, we explore the regulation mechanism of mouse ORMDL3 (mORMDL3) gene in the NIH3T3 system, and find that there are differences between human and mouse ORMDL3 gene expression regulation mechanisms. Furthermore, signaling pathway cAMP/PKA/CREB plays an important role in regulating ORMDL3 expression.

## Materials and Methods

### Cell Culture and Chemicals

Mouse fibroblast cell line NIH3T3 and human type II alveolar lung epithelium cell line A549 were obtained from the American Culture Collection (ATCC). Cells were maintained in Dulbecco’s modified Eagle’ medium (Hyclone) plus 10% heat inactivated fetal bovine serum (FBS), supplemented with penicillin (100 unit/ml) and streptomycin (100 µg/ml). Cells were maintained in an incubator at 37°C and equilibrated with 5% CO_2_ and subcultured using standard cell culture techniques. Forskolin (Sigma-Aldrich, St. Louis, MO), a diterpene obtained from coleus forskohlii, to elevate intracellular cAMP concentration. The protein kinase A (PKA)-specific inhibitor H-89 was also purchased from Sigma-Aldrich. Both forskolin and H-89 were dissolved in dimethyl sulfoxide (DMSO).

### Plasmids

PCR products of mORMDL3 promoter were cloned into the pGL3-Basic vector (Promega, Madison, WI, USA), yielding the promoter reporter plasmids. Substitution mutation and deletion constructs were generated by site-directed mutagenesis kit (Takara). The software TFSEARCH ver.1.3. was used to analyze all possible binding sites on the positive and negative chain of the mORMDL3 promoter, and to ensure the site-directed mutagenesis would not surplus create new binding sites of other transcription factors. All plasmids were verified by sequencing. Names of plasmids and corresponding olignucleotides utilized see Table 1. and Table 2. For overexpression studies, the CREB expression plasmid Y/F-CREB was kindly provided by Dr. M. Montminy (The Salk Institute, CA, USA), and the corresponding control plasmid pcDNA3 was purchased from Invitrogen (Carlsbad, CA, USA).

**Table 1:** Primers used for cloning the mORMDL3 promoter.

Plasmids	Primer sequence (5′–3′) : sense+antisense
pGL3-1129	sense 5′**-**CGG**GGTACC**TTCAGTCCGTCGCGCATATACAG-3′+antisense 5′-GGA**AGATCT**CGATGCCACCCGTCTGGACTCAC-3′
pGL3-590	sense 5′-CGG**GGTACC**GCCAGACCTACACCAGCAAGGATTA-3′+antisense 5′-GGA**AGATCT**CGATGCCACCCGTCTGGACTCAC-3′
pGL3-450	sense 5′-CGG**GGTACC**GGGCAAAGGGTAGTGTTTCTCGGC-3′+antisense 5′-GGA**AGATCT**CGATGCCACCCGTCTGGACTCAC-3′
pGL3-136	sense 5′-CGG**GGTACC**TAGACCAGGGAAAAGCCTACAACTC-3′+antisense 5′-GGA**AGATCT**CGATGCCACCCGTCTGGACTCAC-3′
pGL3-74	sense 5′-CGG**GGTACC**AGAAACTACACTTCCCAAGAGGC-3′+antisense 5′-GGA**AGATCT**CGATGCCACCCGTCTGGACTCAC-3′

Restriction endonuclease sequences are shown in bold.

**Table 2:** Primers used for mutagenesis at the mORMDL3 promoter.

Plasmids	Primer sequence (5′–3′): sense+antisense
mut-STAT6	sense 5′-ACGTGATAGGCGCGTGGTAGTGAC-3′+antisense 5′-TGTGCTCATGGCTAGTGTAGTTTCT-3′
del-STAT6	sense 5′-GCACAACGTGATAGGCGCGTGGTAG-3′+antisense 5′-GTGTAGTTTCTGGTACCTATCGATA-3′
mut-GATA-1	sense 5′-GTAGTGACGTCACCGCCCCG -3′+antisense 5′-CACGCGCCTTATGCGTTGTGCCTCTTGGGAA -3′
del-GATA-1	sense 5′-GCGTGGTAGTGACGTCACCGCCCCGGCTCG-3′+antisense 5′-TTGTGCCTCTTGGGAAGTGTAGTTT-3′
mut-CRE	sense 5′-CGCCCCGGCTCGGGGAGCTGATTCGGCCAGA-3′+antisense 5′-GTACGGTCACTACCACGCGCCTATCACG-3′
del-CRE	sense 5′-CCGCCCCGGCTCGGGGAGCTGATTCGGCCA-3′+antisense 5′-TTGTGCCTCTTGGGAAGTGTAGTTT-3′

### Double-stranded Small Interfering RNA (siRNA)

RNA interference strategy was employed to silence endogenous CREB in NIH3T3 cells. Three kinds of double-stranded siRNAs specific for CREB and one control siRNA were synthesized and high-performance purified (GenePharma). Equimolar amounts of the three individual oligonucleotides specific for CREB were combined before use. The siRNA sequences used are the following (sense) : 5′-GAUUCACAGGAGUCUGUGGtt-3′, 5′-UACAGCUGGCUAACAAUGGtt-3′ and 5′-CCAAGUUGUUGUUCAAGCUtt-3′ for CREB; 5′-UUCUCCGAACGUGUCACGUtt-3′ for control siRNA.

### Transient Transfections and Luciferase Assays

Transient transfections were carried out in NIH3T3 cells and A549 cells using Lipofectamine ™ 2000 (invitrogen) according to the manufacturer’s suggestion. Cells were seeded to 96-well plates (1.5×10^4^ cells per well), after cultured overnight, cells were cotransfected with 200 ng of the firefly luciferase reporter plasmids and 4 ng of the pRL-TK plasmid (Promega) as an internal control for transfection efficiency. For CREB siRNA or CREB overexpression responses, 100 ng luciferase reporter plasmids and 4 ng pRL-TK plasmid with the presence of siRNA for CREB or CREB expression plasmids were cotransfected into NIH3T3 cells, and cells were harvested after 24 hours. Luciferase activity was measured using the Dual Reporter assay system (Promega) and TD-20/20 Turner Designs luminometer. Results were representative of at least three independent experiments.

### Electrophoretic Mobility Shift Analysis (EMSA)

Nuclear extracts were prepared using a Nuclear Extraction Kit (Pierce), according to the manufacturer’s protocol. Three double-stranded oligonucleotides were synthesized (Invitrogen): wild-type 5′-biotinylated double-stranded oligonucleotides containing the CREB-binding motif (TGACGTCA) and the corresponding unlabeled cold competitor are: 5′-CGTGGTAGTGACGTCACCGCCCCG-3′ and the complement. The mutated unlabeled oligonucleotides contained a three nucleotide substitution are: 5′-CGTGGTAGTGACCGTACCGCCCCG-3′ and the complement. Detection of the CREB oligonucleotide complex was performed using the Light Shift Chemiluminescent EMSA Kit (Pierce). Briefly, 10 µg nuclear protein extract was incubated in a buffer containing 10 mM Tris, 50 mM KCl, 1 mM DTT (pH 7.5), 7.5% glycerol, and 75 ng/µl poly(dI-dC) for 20 min on ice. Then 200 fmol of the biotinylated oligonucleotides were added and the reaction mixture was allowed to incubate at room temperature for another 20 min. For competition experiments, unlabeled probes or mutated probes were added to the reaction mixture 20 min before addition of the labeled probe. Finally, the DNA-protein complexes were resolved on a 6% polyacrylamide gel in 0.5×TBE buffer for 1 h.

### Chromatin Immunoprecipitation (ChIP) Assay

ChIP assays were performed with the Chip-IT kit (Active Motif) following the manufacturer’s instructions. A total of 4.5×10^7^ cells were fixed in 1% formaldehyde at room temperature for 15 min. Isolated nuclei were lysed followed by chromatin shearing with the Enzymatic Shearing kit (Active Motif). The chromatin was then immunoprecipitated with anti-RNA Pol II antibody (Active Motif), anti-IgG antibody (Active Motif) and anti-CREB antibody (Santa Cruz). After reverse cross-linking and DNA purification, DNA from input (1∶10 diluted) or immunoprecipitated samples were assayed by PCR, and the products were separated by 3% agarose gel electrophoresis. The primers used for ChIP analysis PCR reaction were: sense: 5′-TAGACCAGGGAAAAGCCTACAACTC-3′; antisense: 5′-GGCCGAATCAGCTCCCCG-3′.

### Quantitative Real-time RT-PCR

RNA extracted from different mouse tissues were kindly provided by Dr. Chao Lu (the First Affiliated Hospital, Nanjing Medical University, China) and ORMDL3 expression pattern was determined by quantitative real-time RT-PCR. Total RNA of NIH3T3 cells were extracted using the Trizol Reagent (Invitrogen) and subsequently reverse transcribed using the PrimeScript RT Master Mix Perfect Real Time kit (TaKaRa). Quantitative real-time RT-PCR was carried out by the Applied Biosystems Step One Plus Real-Time PCR System, using cDNA and SYBR Green (TaKaRa) detection. Expression of the gene of interest was normalized to β-actin and relative expression was calculated using the comparative Ct method. Primers for PCR are following:

ORMDL3: sense: 5′-GGGGGTGGTCAGGAAAGAGGCT-3′;

antisense: 5′-GGGTTGCCAGGAAGCCCACAAA-3′;

β-actin: sense: 5′-AACAGTCCGCCTAGAAGCAC-3′;

antisense: 5′-CGTTGACATCCGTAAAGACC-3′.

### Western Blot Analysis

Cells were harvested by scraping, washed in ice-cold PBS and lysed with lysis buffer containing a protease inhibitor cocktail (Roche). Protein concentrations were determined using the Bio-Rad Protein Assay (Bio-Rad). Protein samples were prepared with 5×SDS sample buffer and loaded at 40 µg of protein per lane for SDS-PAGE. Western blot was performed with ORMDL3 (ab1076389, Abcam), P-CREB and CREB (sc-101663 and sc-58, Santa Cruz) antibodies, followed by anti-rabbit IgG conjugated with HRP. β-Tubulin was detected as loading control. Chemoluminescence signals were quantified using an ECL imager, and analyzed using Quantity One software (Bio-Rad).

### Statistical Analysis

Statistical analysis was done using SPSS Software (version 16.0). Results were analyzed using paired or unpaired t tests, and *P*<0.05 was considered significant.

## Results

### ORMDL3 is Wildly Expressed in Mouse Tissues

**Figure 1:**
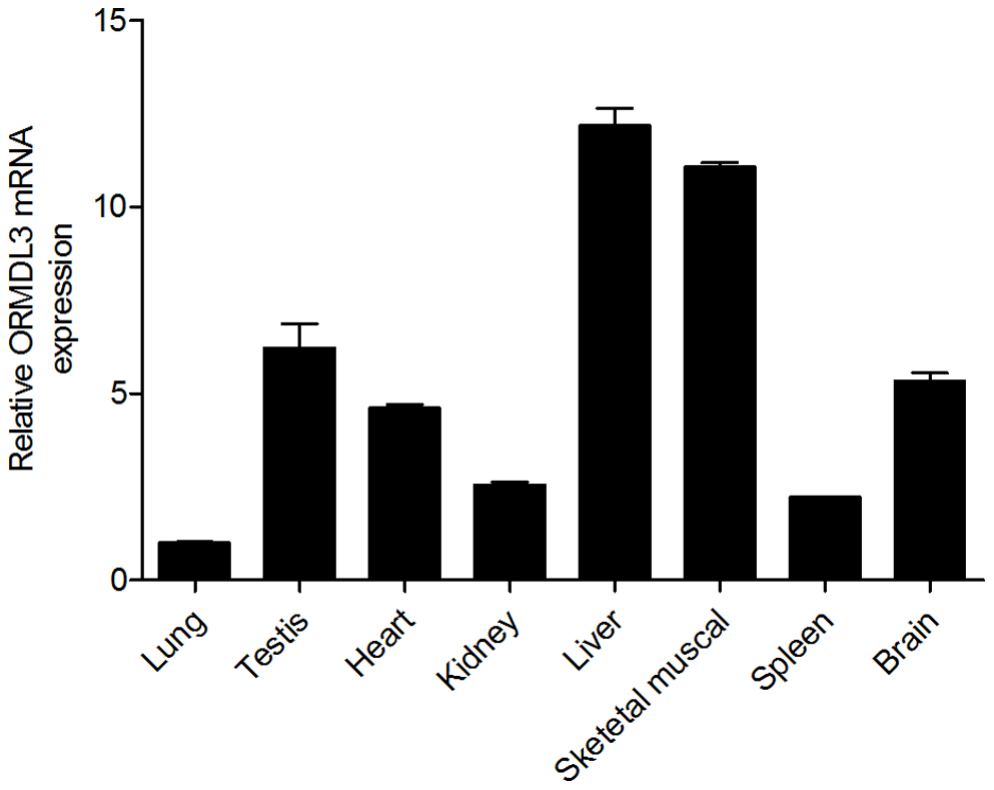
Quantitative Real-time RT-PCR analyses of expression pattern of mouse ORMDL3 gene. ORMDL3 expression in lung is normalized to 1, and the other organs are compared relative to the normalized lung level.

To study the expression pattern of mORMDL3 gene, quantitative real-time PCR was performed. PCR expression analysis on six weeks male BALB/c mice tissue samples showed a ubiquitous expression pattern of mORMDL3 gene (Fig. 1). The basal expression of mORMDL3 was relatively high in liver and skeletal muscle but low in lung.

### Region Located between −74 and +141 Contributes to the Basal Activity of mORMDL3 Promoter

**Figure 2:**
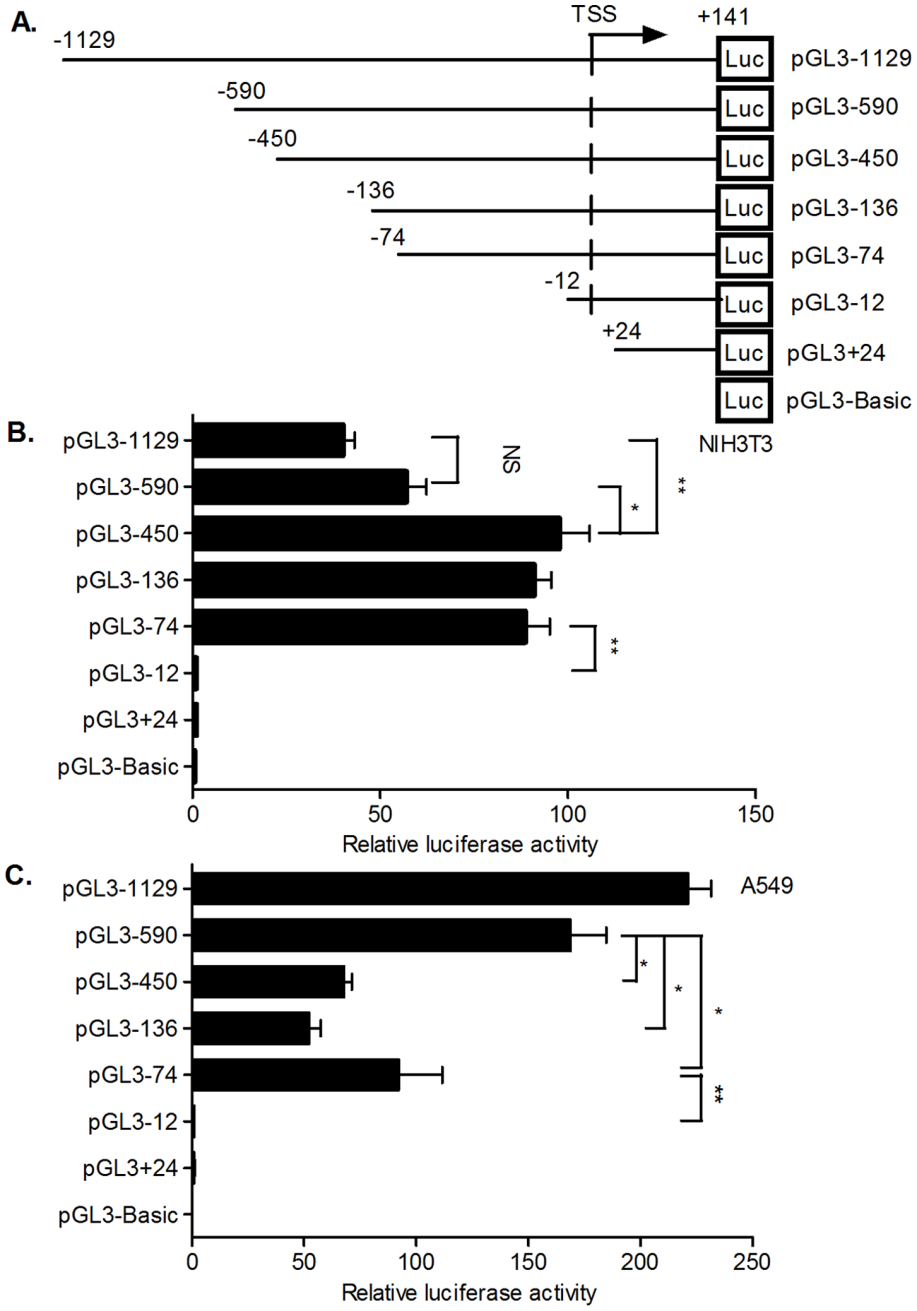
Functional analysis of the mouse ORMDL3 promoter. (A) Different sizes of 5′ deletion fragments of mouse ORMDL3 promoter were cloned into pGL3-Basic luciferase reporter plasmids. The numbering is relative to the TSS. The reporter constructs were transfected into NIH3T3 cells (B) and A549 cells (C) and the reporter activities were measured. Relative firefly luciferase activities were averages of three independent transfections normalized to renilla control activities. Data are presented a mean ± S.D. (*P<0.05, **P<0.01).

To identify the functional proximal promoter of mORMDL3 gene and locate the key genomic regions involved in mORMDL3 gene expression, basing on the National Center for Biotechnology Information (NCBI) sequence database, we defined the reported mORMDL3 (NM_025661.4) 5′ end position as the transcriptional start site (TSS) reference, designated as position +1. Compared to this, we cloned a series of luciferase reporter plasmids containing different mORMDL3 promoter truncations (Fig. 2A). These constructs were transiently transfected into NIH3T3, and promoter activity was assessed by measuring luciferase activity.

As shown in Fig. 2B, luciferase assays revealed a 69-fold increase in promoter activity of pGL3-1129 compared to the empty vector pGL3-Basic in NIH3T3 cells, indicating a functional promoter was contained in the region of −1129/+141 bp of the mORMDL3 gene. Progressive mORMDL3 promoter truncations revealed that deleting the sequence between −1129 and −590 has no significant affect on luciferase expression, as pGL3-590 showed a luciferase activity similar to pGL3-1129. In contrast, the luciferase activity of pGL3-450 was 1.7-fold of pGL3-590 (*P*<0.05), suggesting that the −590/−450 sequence contained negative regulatory elements. There was no significant change in transcriptional activity when it was further deleted to position −136 or −74. However, when the promoter was deleted to position −12 (pGL3-12) or +24 (pGL3+24), transcriptional activity decreased dramatically, which was similar to pGL3-Basic.

To explore whether those differences among the mORMDL3 promoter truncations were cell specific, we also transfected these plasmids into A549 cells (Fig. 2C). Like in NIH3T3 cells, there was no significant transcriptional activity difference between pGL3-1129 and pGL3-590. However, deleting the region between −590 and −450 resulted in a reduction in transcriptional activity, which differed from the observation in NIH3T3 cells. Meanwhile, it was worth noting that all the tested mORMDL3 promoter constructs, containing −74/−12 fragments, were capable of inducing a significant increase in luciferase activity compared with pGL3-Basic. On the contrary, activities of constructs lack the region −74/−12 (pGL3-12 and pGL3+24) almost close to pGL3-Basic both in NIH3T3 cells and in A549 cells.

The above results indicated that the functional proximal minimal promoter of mORMDL3 gene was spanning from −74 to +141 relative to the TSS, and the region −74/−12 may contain important regulation elements, which may play pivotal roles in keeping basal transcriptional activity of mORMDL3 gene.

### CREB Transactivates mORMDL3 Through CRE in the mORMDL3 Promoter

**Figure 3:**
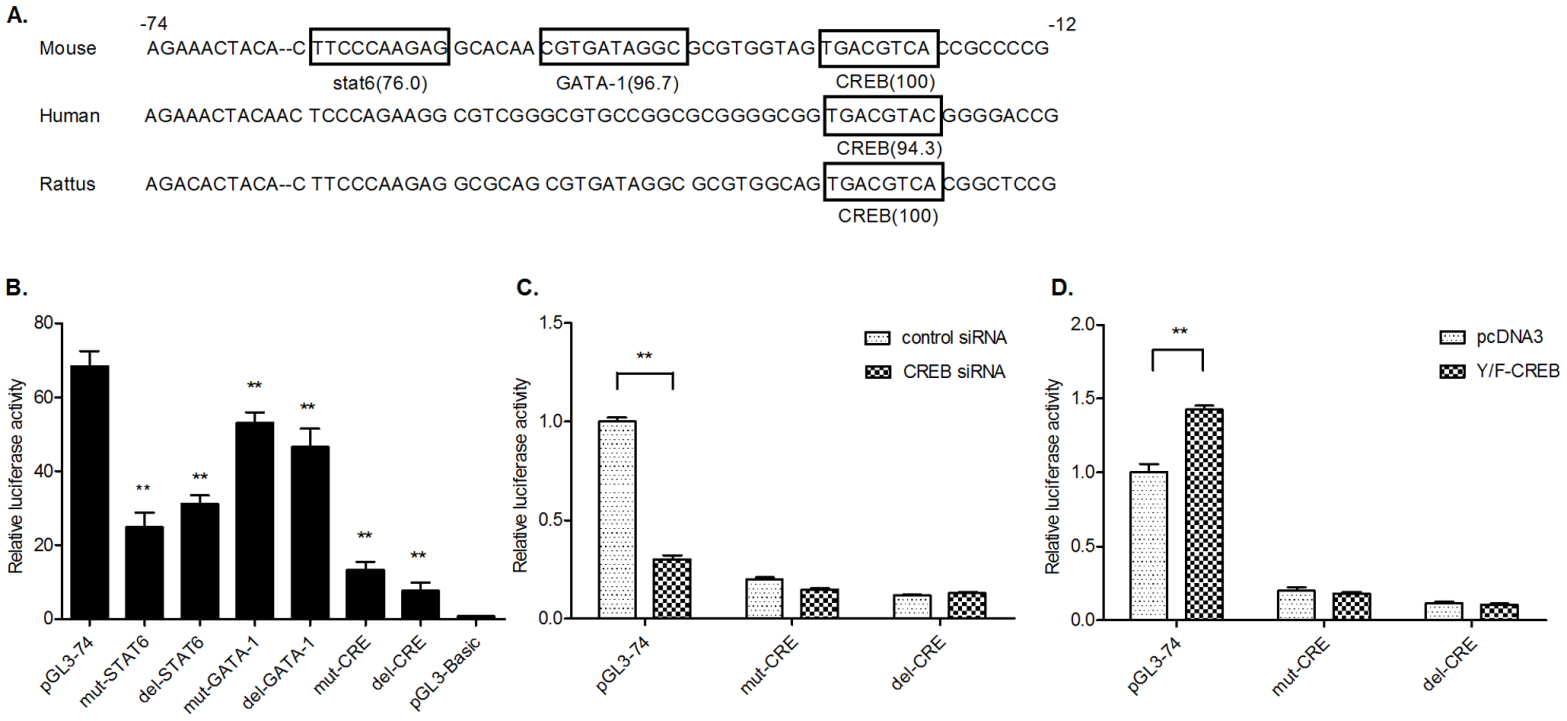
Characterization of the transcription factor binding elements. (A) Nucleotide sequence of the proximal minimal promoter of the mouse ORMDL3 gene and the corresponding sequences of human and rattus. The numbering of the sequence is relative to the TSS. Putative transcription factor binding sites were underlined and the numbers in brackets represent the homology they shared with their corresponding consensus sequence. (B) NIH3T3 cells were transfected with the ORMDL3 promoter (pGL3-74), three nucleotide substitution mutant variants (mut-STAT6, mut-GATA-1 and mut-CRE), three site-deletion mutant variants (del-STAT6, del-GATA-1 and del-CRE) and the promoter-less pGL3-Basic construct. Relative luciferase activity expressed as the fold induction relative to pGL3-Basic vector. Results are presented as mean ± S.D. of three independent experiments (** *P*<0.01 vs. wild type pGL3-74). (C-D) pGL3-74, mut-CRE or del-CRE was transfected into NIH3T3 cells with the presence of control siRNA, CREB siRNA, pcDNA3 vector or Y/F-CREB. Relative luciferase activity of pGL-74 cotransfected with pcDNA3 empty vector or control siRNA was set as 1(*P<0.05 vs. control, **P<0.01 vs. control).

The sequence from −74 to −12 upstream of the TSS, contained in the functional proximal promoter of mORMDL3, was analyzed using online software TFSEARCH ver.1.3 (Fig. 3A). Two highly conserved protein-binding sequence for transcription factor GATA-1 and CREB were predicted, and the homology they shared with their corresponding consensus sequences were 96.7% and 100%, respectively. Previous report indicated that ORMDL3 expression could be facilitated by Th2 cytokine (IL-4 and IL-13)-induced STAT-6 in the lung of mice, in particular in airway epithelial cells, and STAT6 may influenced ORMDL3 expression indirectly as there was no predicted transcription factor binding site for STAT6 in region up to 4,000 bp 5′ of the transcriptional start site of mORMDL3 [9]. Nevertheless, in our analysis, we found an STAT6 binding site in the proximal promoter of mORMDL3, but this STAT6-site shared only 76.0% homology with its consensus motif. On the contrary, the consensus sequence (TGACGTCA) for CREB at position −27 to −20, which share 100% homology with the consensus cyclic AMP-responsive element (CRE), was highly conserved in mouse, rattus, and human beings (Fig. 3A).

To determine the functions of these binding sites, we carried out site-direct mutagenesis (point mutation, mut- or site-direct deletion, del-) in the wild plasmid pGL3-74. We transfected these constructions into NIH3T3 and measured the luciferase activities 24 h later. As shown in Fig. 3B, point mutation (nucleotide substitution) of the STAT6 binding site (mut-STAT6, TTCCCAAGAG→TAGCCATGAG) or STAT6-site deletion mutation (del-STAT6) resulted in 63% and 54% decrease respectively in the promoter activity compared with the context of intact pGL3-74. Point mutation and deletion mutation of the GATA-1 binding site (mut-GATA-1 (CGTGATAGGC→CGCATAAGGC) and del-GATA-1) dropped the promoter activity by 22% and 32% respectively, whereas CRE-site mutations (mut-CRE (TGACGTCA→TACGGTCA) and del-CRE) led to 80% and 88% reduction in the ORMDL3 promoter activity compared to that of the wild type.

As mutation analysis showed the binding site for CREB (CRE-site) play a dominant role, we tried to explore the effect of CREB on pGL3-74 luciferase activity. CREB siRNA or CREB expression vector Y/F-CREB was transfected into NIH3T3 cells. Knocking down endogenous CREB reduced the luciferase activity of pGL3-74 by 70%, and this reduction could be abrogated by mutating the CRE-site, as luciferase activities of mut-CRE and del-CRE were not significantly changed at the presence of CREB siRNA (Fig. 3C). Y/F-CREB is a gain-of functional mutant containing a Tyr134Phe mutation, which makes CREB behave as a constitutive activator in vivo, stimulating target gene expression like wild type CREB [17]. As shown in Fig. 3D, overexpression of CREB enhanced the luciferase activity of pGL3-74 by 40%, while mut-CRE and del-CRE failed to response to Y/F-CREB. All the results above suggested that the CRE-site is an important positive regulatory element in the mORMDL3 promoter, and transcription factor CREB regulates mORMDL3 promoter activity through this site.

### CREB Binds to the mORMDL3 Promoter in vitro and in vivo

**Figure 4:**
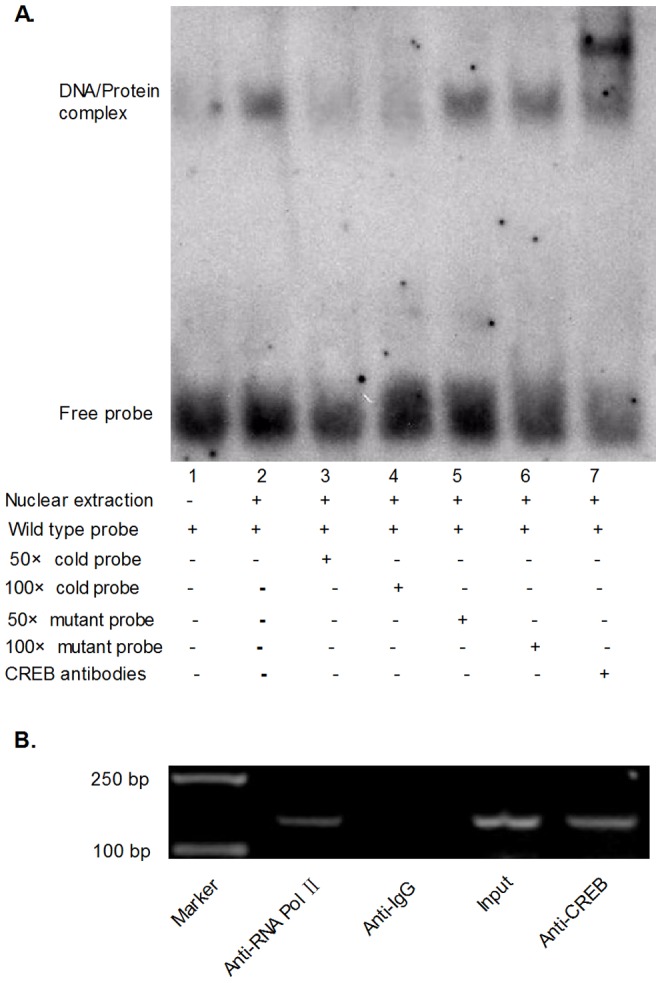
CREB specifically binds to the promoter of mouse ORMDL3. (A) EMSA assays showing direct binding of CREB to the ORMDL3 promoter in vitro. Nuclear protein extracts from NIH3T3 cells were incubated with labeled wild type probe containing CREB binding site in presence of 50×cold probe (lane 3), 100×cold probe (lane 4), 50×mutant probe (lane 5), 100×mutant probe (lane 6) or absence of any competitor (lane 2). The super-shift assay conducted using 20 ng anti-CREB antibodies (lane 7). (B) ChIP assays to test the binding of CREB to the promoter of ORMDL3 in vivo. Anti-RNA Pol II (positive control antibody) and anti-CREB bodies precipitated proteins bound in vivo to the amplified sequence of the endogenous ORMDL3 promoter whereas non-specific IgG (negative control antibody) failed to precipitate proteins bound in vivo this sequence. PCR products were 145 bp and were visualized by agarose gel electrophoresis and ethidium bromide staining.

CREB as a transcription factor, when activated can bind to the promoter regions of its target genes which contain CRE sites TGACGTCA, or CRE half sites CGTCA/TGACG, and then mediate transcription. To validate whether CREB binds to the mORMDL3 promoter, we performed electrophoretic mobility shift assay. As shown in Fig. 4A, the nuclear protein from NIH3T3 cells bound to the labeled wild type oligonucleotide and formed the protein-DNA complex (lane 2, Fig. 4A). Competition assays (using 50, 100-fold excess of cold competitor oligonucleotides) verified the specificity of the CREB/DNA interaction (lanes 3 and 4, Fig. 4A), whereas the mutant probe (using 50, 100-times as much as wild type probe) had no effect on the formation of protein-DNA complex (lanes 5 and 6, Fig. 4A). Further super-shift study shown in the last lane, the complex was super shifted when incubated with CREB-antibody (lane 7, Fig. 4A). ChIP assay was also performed to examine whether CREB interacts with promoter in vivo. The chromatin prepared from the NIH3T3 cells were immunoprecipitated with anti-RNA Pol II, anti-IgG and anti-CREB antibodies. Then, the DNA precipitated in the complexes were subjected to PCR with primers flanking the region containing the CRE-site. As shown in Fig. 4B, anti-RNA Pol II and anti-CREB antibodies precipitated proteins bound in vivo to the amplified sequence of the mORMDL3 promoter, whereas non-specific IgG antibody (negative control antibody) failed to precipitate proteins bound in vivo this sequence, suggesting that CREB can bind to the proximal promoter of mORMDL3 in vivo. Together, CREB transcription factor is capable of binding to the mORMDL3 promoter region.

### CREB Up-regulates mORMDL3 mRNA and Protein Expression

**Figure 5:**
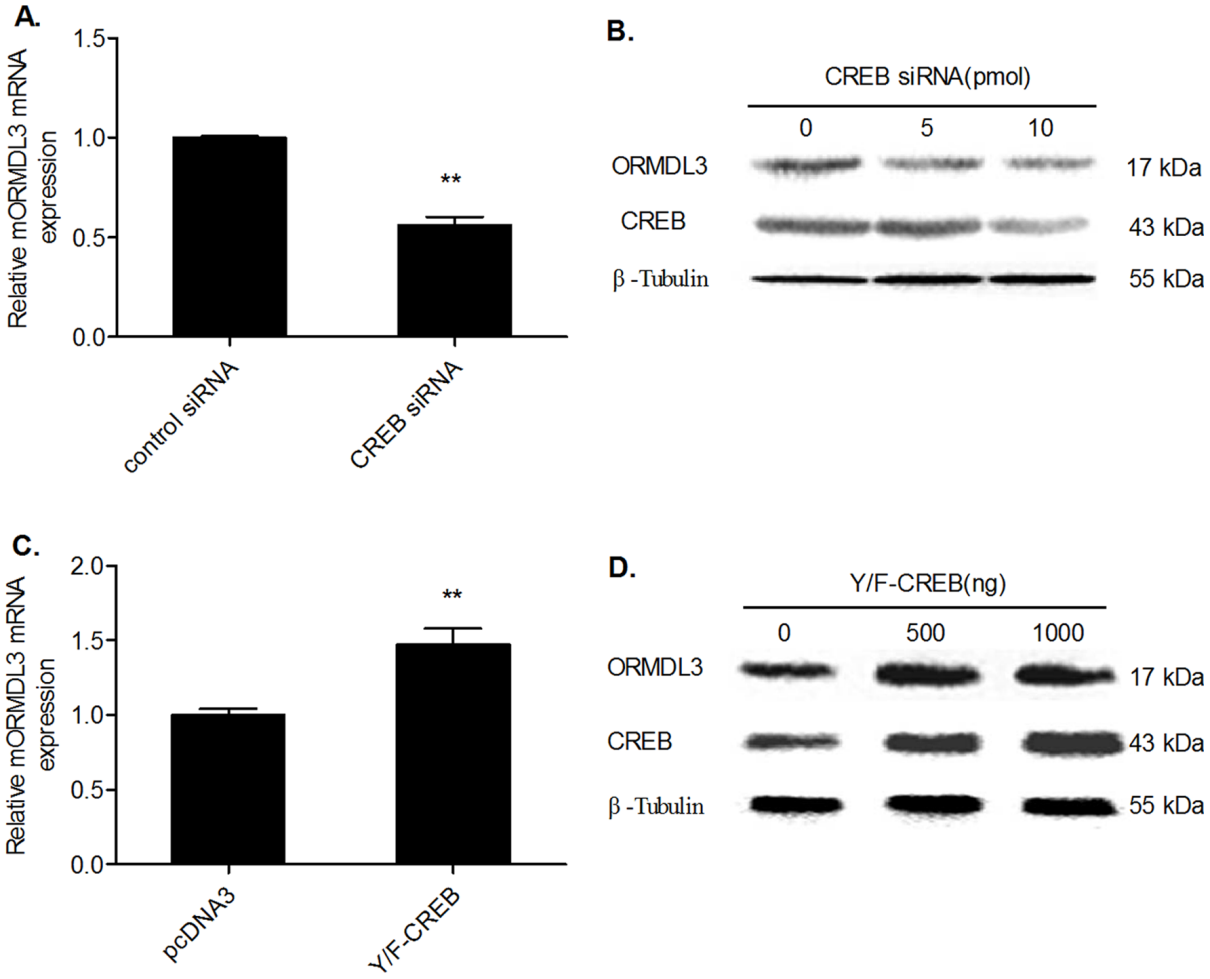
CREB regulates the expression of ORMDL3 in NIH3T3 cells. NIH3T3 cells were transiently transfected with siRNA against CREB (CREB siRNA), negative control siRNA, pcDNA3 empty vector or Y/F-CREB. (A and C) ORMDL3 mRNA was measured by real-time PCR 24 h after transfection and results were normalized toβ-actin. Data are representative of three independent experiments (**P<0.01). (B and D) Interference efficiency, overexpression efficiency and ORMDL3 protein expression were analyzed by Western blot with anti-CREB, anti-ORMDL3 and anti-β-Tubulin antibodies.

To further explore the role of CREB in regulating mORMDL3 expression, CREB siRNA or CREB expression vector (Y/F-CREB) was transfected into NIH3T3 cells. mORMDL3 mRNA and protein levels were detected by real time RT-PCR and Western blotting, respectively. As illustrated in Fig. 5, RNAi-mediated 62% knockdown of CREB resulted in a reduction of mORMDL3 transcript level by 43% and mORMDL3 protein level by 59%. In contrast, overexpression of CREB by 1.53-fold resulted in a 1.47-fold more mORMDL3 mRNA level than the negative control pcDNA3 and a concomitant increase of 45% in the mORMDL3 protein level.

### CAMP/PKA/CREB Signal Way Promotes mORMDL3 Expression

**Figure 6:**
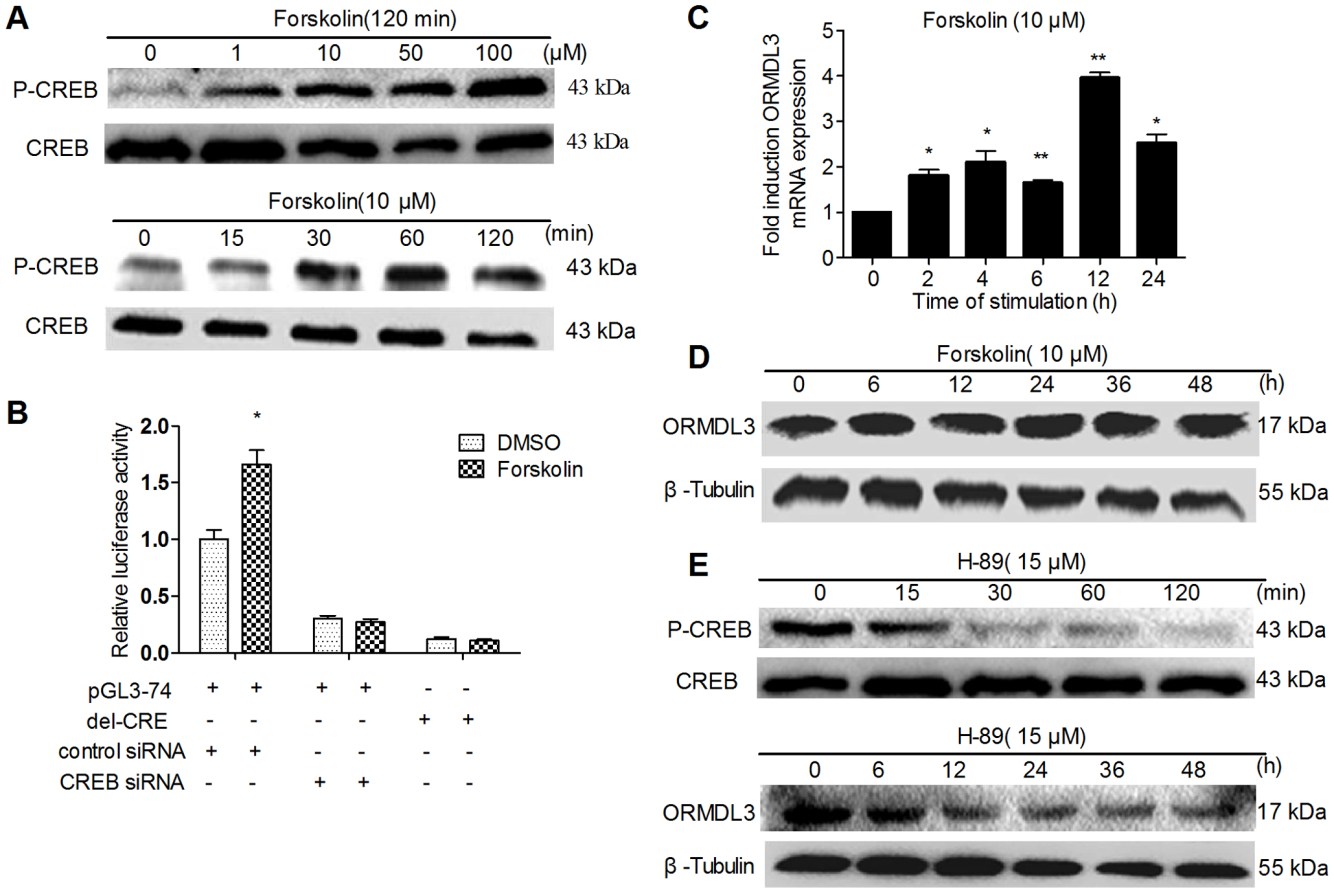
CAMP/PKA/CREB signal way regulates mORMDL3 expression in NIH3T3 cells. (A) Dose response and time course for forskolin-induced CREB phosphorylation. (B) CREB mediates forskolin-induced ORMDL3 promoter (pGL3-74) activity. (C) Real Time RT-PCR analyzes the regulation of ORMLD3 transcription in response to forskolin. Data are normalized toβ-actin and are representative of three independent experiments (*P<0.05 vs. untreated, **P<0.01 vs. untreated). (D) Western blot analyze the regulation of ORMLD3 protein expression in response to forskolin. (E) Western blot analyze the inhibitory effect of H-89 on CREB phospholation and on ORMDL3 protein expression.

Previous studies indicated that the PKA pathway was responsible for immediate stimulation of CREB [18]. As shown in Fig. 6A, incubation of forskolin, which elevates intracellular cAMP concentration, an activator of PKA, resulted in increased phosphorylation of CREB in NIH3T3 cells in both dose-dependent and time-dependent manner. We co-transfected pGL3-74 with control siRNA or CREB siRNA into NIH3T3 and incubated for 24h, then further incubated for 6 h at the presence of forskolin (10 µM). Luciferase analysis (Fig. 6B) showed that forskolin led to an increase promoter activity of pGL3-74, while the presence of CREB siRNA abrogated the forskolin’s enhancement. Besides, promoter construction without CRE-site (del-CRE) failed to response to forskolin. As for mORMDL3 mRNA induction (Fig. 6C), forskolin incubation induced mORMDL3 mRNA expression was already 1.8-fold after 2 h, 1.6-2.1-fold after 4–6 h, reached a peak of 4.0-fold after 12 h and declined to 2.5-fold after 24 h. However, the protein level of mORMDL3 increased slowly after forskolin induction, and the inducible effects reached a peak of 2.0-fold after 24 h and 48 h, respectively (Fig. 6D). To further confirm the effects of PKA/CREB signal way on mORMDL3 expression, we applied H-89, an inhibitor of PKA [18]. As shown in Fig. 6E, incubation of H-89 led to a decrease in phosphorylation of CREB, and the inhibitory effect of H-89 on mORMDL3 protein expression was already significant after 6 h incubation, and was most obvious at 12 h, then kept steady. Collectively, cAMP/PKA regulate mORMDL3 expression, and this effect may partly mediated by CREB.

## Discussion

Considerable evidence supports the deduction that ORMDL3 is associated with the development of asthma. We previously reported that ORMDL3 mRNA level in the peripheral blood of recurrent wheeze patients are higher than control group. Transcription regulation research showed that ORMDL3 expression is cooperatively regulated by transcription factors Ets-1, p300 and CREB [13]. Miller et al. demonstrated that ORMDL3 expression can be significantly induced by allergen in airway epithelial cells of mice, and IL-4/IL-13/STAT6 signal way plays a vital role in inducing mORMDL3 expression [9]. Here, we characterized the promoter of mORMDL3 and found that there were some differences in transcriptional regulation mechanisms of ORMDL3 gene between human and mouse despite ORMDL3 exhibiting 96% identity between man and mouse.

In human tissues, ORMDL3 gene showed a ubiquitously expression pattern in adult and fetal samples with some minor tissue-specific differences [1], [2]. In our study, mouse ORMDL3 gene was also wildly expressed in BALB/c mice samples with slightly differences. And both in human and mouse tissues, basal transcription level of ORMDL3 in lung are relatively low.

It is well known that CREB activates transcription following phosphorylation of serine 133 residue in the activation domain by several kinases and in response to a variety of stimuli [19]. In this study, we demonstrated that CREB could bind to the promoter of mORMDL3 and regulate the basal transcription of mORMDL3. In somatic tissues, TATA boxes downstream of CREs are required for robust transcriptional induction by CREB [20]. Four putative TATA boxes (TFSEARCH ver.1.3. threshold score:75.0) were found between −1129 and −590 bp relative to TSS while no TATA box was found in the functional proximal minimal promoter of mORMDL3 gene. But our data indicates that CREB plays a pivotal role in activating the basal transcription of mORMDL3, which is agree with the fact that the presence of a TATA element is not a pre-requisite for regulation by CREB [19].

To our knowledge, many genes contain TATA-less promoters are thus enriched in GC and often regulated by Sp1 [21]. As for mORMDL3 gene, the proximal minimal promoter contains no TATA box, but has a CpG island which possesses a high GC content (67.9%) with an observed CpG/expected CpG ratio (ObsCpG/ExpCpG) of 0.928, as calculated by CpG island searcher (http://cpgislands.usc.edu/). Meanwhile, according to the defining criteria for a CpG island requires a minimum 200-bp stretch of DNA with a C+G content of 50% and an ObsCpG/ExpCpG in excess of 0.6 [22], the proximal minimal promoter region of mORMDL3 may contain a functional CpG island. However, whether epigenetic regulation or Sp1 take part in the regulating of ORMDL3 expression is yet to be confirmed.

Here, we also show that cAMP-dependent protein kinase (PKA) can phosphorylate CREB and activate CREB-mediated mORMDL3 transcription. To our knowledge, agonists activating β_2_-adrenoceptors (β_2_ARs) on airway smooth muscle are the drug of choice for rescue from acute bronchoconstriction in patients with asthma, and the presumed cellular mechanism of action involves the generation of intracellular cAMP, which can activate the effector molecules PKA and in turn antagonize airway smooth muscle contraction, proliferation and migration [23]. From this perspective, our results invite consideration as to how the effect of current major therapy β- agonists could be suboptimal when administered against a background of up-regulated ORMDL3. Prior report showed that ORMDL3 binds and inhibits the sarco-endoplasmic reticulum Ca^2+^ pump (SERCA) resulting in a reduced ER Ca^2+^ concentration and increased the unfolded-protein response (UPR), a process considered as an endogenous inducer of inﬂammation [16]. Besides, previous work reported that cAMP-elevating agents indirectly modulate Ca^2+^ homeostasis through phospholamban, which inhibits SERCA activity [24]. Thus, we speculate that up-regulating ORMDL3 expression may be another way that cAMP-elevating agents indirectly inhibit SERCA activity. Notably, airway smooth muscle cells from patients with asthma already have decreased level of SERCA. siRNA-mediated knockdown of SERCA in airway smooth muscle cells from healthy subjects increased cell spreading, eotaxin-1 release and proliferation which implicating that a deficiency in SERCA in airway smooth muscle cells in asthma may play a key role in mechanisms of airway remodeling [25], [26]. Thus, ORMDL3 high-expression may also contribute to airway remodeling in patients suffering from asthma or mice model sensitized with allergen. Besides, cAMP-elevating agents may play a role in aggravating airway remodeling since cAMP/PKA/CREB signal way up-regulates ORMDL3 expression in this study.

In summary, our study reveal that the mouse ORMDL3 gene is wildly expressed in mice as it is in man, and the functional proximal minimal promoter is located within a region of 215 bp at position −74/+141 relative to the TSS. CRE site is crucial for the basal transcription of mORMDL3 promoter. Transcription factor CREB regulates ORMDL3 transcription by directly binding to this CRE-site and kinase PKA can induce mORMDL3 expression through phosphorylating CREB.
